# Adequacy of inhale/exhale breathhold CT based ITV margins and image-guided registration for free-breathing pancreas and liver SBRT

**DOI:** 10.1186/1748-717X-9-11

**Published:** 2014-01-09

**Authors:** Wensha Yang, Benedick A Fraass, Robert Reznik, Nicholas Nissen, Simon Lo, Laith H Jamil, Kapil Gupta, Howard Sandler, Richard Tuli

**Affiliations:** 1 Department of Radiation Oncology, Cedars Sinai Medical Center, Los Angeles, CA 90048, USA; 2 Department of Surgery, Cedars Sinai Medical Center, Los Angeles, CA 90048, USA; 3 Department of Medicine, Cedars Sinai Medical Center, Los Angeles, CA 90048, USA

**Keywords:** SBRT, Pancreas, Liver, Fiducial, Radiotherapy, Stereotactic body radiation therapy

## Abstract

**Purpose:**

To evaluate use of breath-hold CTs and implanted fiducials for definition of the internal target volume (ITV) margin for upper abdominal stereotactic body radiation therapy (SBRT). To study the statistics of inter- and intra-fractional motion information.

**Methods and materials:**

11 patients treated with SBRT for locally advanced pancreatic cancer (LAPC) or liver cancer were included in the study. Patients underwent fiducial implantation, free-breathing CT and breath-hold CTs at end inhalation/exhalation. All patients were planned and treated with SBRT using volumetric modulated arc therapy (VMAT). Two margin strategies were studied: Strategy I uses PTV = ITV + 3 mm; Strategy II uses PTV = GTV + 1.5 cm. Both CBCT and kV orthogonal images were taken and analyzed for setup before patient treatments. Tumor motion statistics based on skeletal registration and on fiducial registration were analyzed by fitting to Gaussian functions.

**Results:**

All 11 patients met SBRT planning dose constraints using strategy I. Average ITV margins for the 11 patients were 2 mm RL, 6 mm AP, and 6 mm SI. Skeletal registration resulted in high probability (RL = 69%, AP = 4.6%, SI = 39%) that part of the tumor will be outside the ITV. With the 3 mm ITV expansion (Strategy 1), the probability reduced to RL 32%, AP 0.3%, SI 20% for skeletal registration; and RL 1.2%, AP 0%, SI 7% for fiducial registration. All 7 pancreatic patients and 2 liver patients failed to meet SBRT dose constraints using strategy II. The liver dose was increased by 36% for the other 2 liver patients that met the SBRT dose constraints with strategy II.

**Conclusions:**

Image guidance matching to skeletal anatomy is inadequate for SBRT positioning in the upper abdomen and usage of fiducials is highly recommended. Even with fiducial implantation and definition of an ITV, a minimal 3 mm planning margin around the ITV is needed to accommodate intra-fractional uncertainties.

## Background

Image guided radiotherapy (IGRT) has improved the accuracy of radiation therapy (RT) by providing 3D imaging registration based on volumetric anatomic information. A linear accelerator (LINAC) treatment machine equipped with cone beam computed tomography (CBCT) can acquire high resolution images with excellent skeletal anatomy contrast and useful soft tissue contrast. This allows for significantly improved registration and tumor targeting accuracy as compared to skeletal anatomy-based registration using either kV or MV portal images. Lung treatments have benefited greatly from the use of CBCT due to low density. On the other hand, many tumor targets do not show sufficient soft tissue contrast using CBCT [1], thereby rendering it ineffective other than for skeletal anatomy registration. However, use of skeletal anatomy only can result in significant geometrical errors. This lack of tumor and soft tissue CT contrast is a prominent problem in the abdominal and pelvic regions, where organs are substantially influenced by both inter- and intra-fractional motion [2,3]. In most conventionally fractionated RT, generous margins have been used to account for the large geometrical uncertainties in the tumor position in this anatomical region.

Increasingly, stereotactic body radiation therapy (SBRT) is being utilized to treat hepatic and pancreatic cancers given the improved local control rates compared to conventionally fractionated RT [4,5]. During SBRT, higher doses per fraction are delivered, thereby increasing the risk of injury to nearby uninvolved critical organs, which then motivates attempts at more aggressive margin reduction strategies. Recognizing the deficiencies in tissue contrast using CBCT for upper abdominal SBRT guidance, a number of clinical investigators have started to implant fiducial markers in abdominal organs, mainly under endoscopic ultrasound (EUS) guidance [6-9]. In addition to allowing more accurate daily tumor targeting, fiducial markers also provide an excellent opportunity to examine inter- and intra-fractional motion of the tumor relative to the skeletal anatomy, with the ultimate aim of minimizing target volume margins in a patient-specific manner. However, published data on this topic are still limited. Varadarajulu et al. reported on inter-fractional discrepancies between skeletal landmark and intra-pancreatic fiducial alignment using kV 2D images [10], however, this snapshot of fiducial position could not differentiate inter-fractional motion from intra-fractional motion. The relative uncertainty associated with predicting inter- and intra-fractional motion during free-breathing SBRT of abdominal tumors can have a significant impact on planning dosimetry and potentially even clinical outcomes.

To overcome this problem, in this work we assess the utility of breath-hold simulation CT scans for motion assessment and free-breathing treatment of pancreatic and liver cancer patients implanted with fiducial markers and guided using CBCT and kV 2D imaging, which samples the probability density motion distribution of the fiducials while patient is breathing. Specifically, we attempt to 1) evaluate commonly used planning target volume (PTV) margin calculation methods for skeletal anatomy and fiducial marker alignment, respectively. The first method is a tight margin on ITV, and the second is a generous margin on GTV; 2) determine whether the internal target volume (ITV) margin can be accurately predicted from the pre-treatment tumor motion range evaluated using inhale and exhale breath-hold CT simulation scans; and 3) the variation in intra- and inter-fractional tumor motion as inferred from the location of implanted fiducial markers.

## Methods and materials

### Fiducial placement, CT-simulation and motion assessment

Eleven consecutive patients with locally advanced pancreatic (n = 7) or liver (n = 4) cancer were studied under an IRB approved protocol. Prior to CT simulation, patients were implanted with 2–5 radio-opaque fiducial markers (Visicoil, IBA; 0.75 × 3 mm) in the periphery of the tumor, with 2–3 fiducials around each lesion under EUS guidance [11]. More than 72 hours after implantation, patients underwent CT simulation and were positioned on a wing board with both arms raised above the head using Vac-Lok™ (MED-TEC, Orange City, IA) bag immobilization.

**Figure 1:**
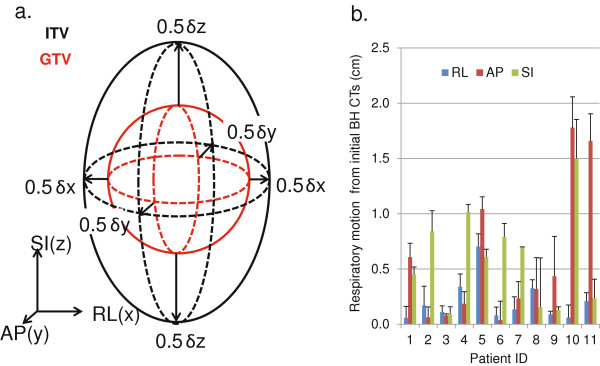
**GTV to ITV margin expansion and respiratory fiducial motion for all patients. a**. GTV(red) to ITV(black( expansion strategy. **b**. The motion range evaluated from breath-hold CTs for 11 patients (1–7 are pancreas patients, 8–11 are liver patients), with averages 0.6 (0–1.8) cm AP, 0.6 (0.1-1.5) cm SI, and 0.2 (0.1-0.7) cm RL.

Patients were scanned from the carina to L5/S1 using a dual slice GE high speed NXi scanner with 2.5 mm slice thickness while free breathing (FB) after receiving oral and intravenous (IV) contrast. They were then instructed to inhale normally and hold their breath while an inhale breath-hold (IBH) scan was performed. Similarly, an exhale breath-hold (EBH) CT was performed. The tumor was first identified on the FB scan and IBH/EBH scans were then taken with images obtained 25 slices superior and inferior to the tumor. The motion range of individual fiducial markers was calculated using the coordinates of the fiducial on the IBH and EBH scans. The average displacements of the fiducials in RL (right- left) δx, AP (anterior-posterior) δy and SI (superior-inferior) δz directions, respectively, were used to create the non-isotropic GTV to ITV margin using the margin tool provided in Eclipse. Figure 1a shows the margin expansion strategy.

### Treatment planning

The GTV was drawn to include the gross primary tumor and enlarged lymph nodes considered suggestive of metastatic involvement on diagnostic imaging (CT and/or PET). The GTV was identified on FB simulation CT scan, which was obtained with both intravenous and oral contrast [12]. Two margin strategies were investigated. In strategy I, the PTV was defined as the ITV with a 3 mm isotropic expansion [13]. This was the clinically implemented strategy based on the availability of fiducial markers for the SBRT daily alignment. All patients were treated with 25–40 Gy in 5 daily fractions. Volumetric modulated arc therapy (VMAT) with 2–3 arcs was used for treatment planning for all patients in the study [14]. Treatment plans were optimized so at least 90 - 100% of the PTV received the prescription dose, with maximum allowable dose of 120%. All plans were optimized to meet the clinically used normal tissue dose constraints including: D_max_ < 8 Gy for cord, D_75%_ < 12 Gy for combined kidneys, mean dose < 12 Gy for liver-GTV, and V_15Gy_ < 9 cc, V_20Gy_ < 3 cc, D_50%_ < 12 Gy, D_max_ < 33 Gy for stomach, duodenum, and small bowel [15]. In strategy II, a 1.5 cm GTV to PTV expansion was used without ITV calculation. Identical prescriptions and normal tissue dose constraints were used. The number of patients who did not meet the SBRT dose constraints was identified.

### Treatment delivery

Prior to each treatment fraction, both CBCT and one pair of kV images, using standard abdominal imaging parameters suggested by Varian, were obtained to verify the position of the tumor. CBCT was first aligned to the FB planning CT using the skeletal anatomy. A second registration was then performed with alignment to fiducials (with a 2 mm tolerance). The relative couch shifts between the skeletal and fiducial registrations after CBCT were used to evaluate the differences in soft tissue displacement (“bone-marker difference”). kV images were then obtained and registered to fiducials after CBCT fiducial shifts. The resulting displacement from CBCT-matching to kV-matching was recorded as CBCT-kV difference.

## Results

All patients tolerated the EUS-guided fiducial placement without any associated morbidity. A total of 33 fiducials were implanted. We noted no migration or loss of markers from time of placement until the end of treatment. As shown in Figure 1b, calculation of the respiratory motion range of fiducial markers from the BH CT images showed the average motion in the RL direction was significantly less than the two other directions. Four patients showed greater than 1 cm motion in one or more directions; two had pancreatic tumors, while 2 had liver tumors. The average ITV margins for the 11 patients were 2 mm RL, 6 mm AP, and 6 mm SI.

**Figure 2:**
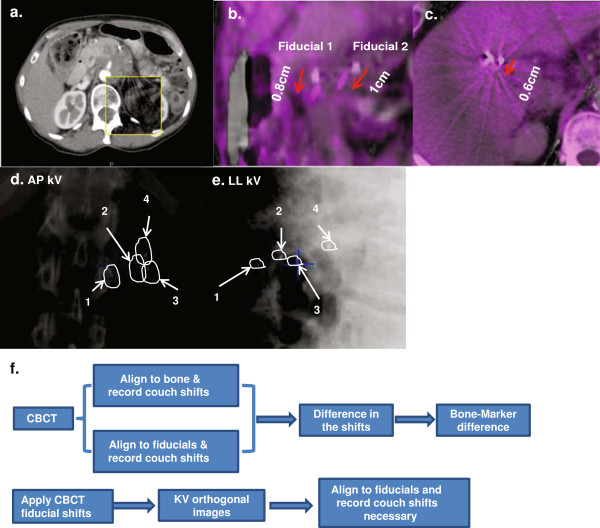
**Example images of fiducial motion and DRR to kV registration; flow chart of pre-treatment image registration. a**. A typical pre-RT CBCT registered to planning CT using skeletal landmarks. **b**. Fiducial displacement from planning CT to CBCT with skeletal alignment for a pancreas patient. **c**. Fiducial displacement from planning CT to CBCT with skeletal alignment for a liver patient. **d-e**. an example of kV orthogonal images with fiducial matching to the contour derived from DRR. **f**. Flow chart describes how the imaging guidance was performed before the treatment and how the shifts were resolved.

**Figure 3:**
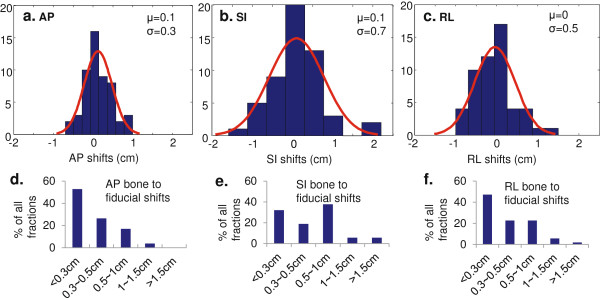
**Statistics for bone to fiducial shifts. a-c**. Histograms of bone-marker discrepancies for AP, SI, and RL directions, respectively. Gaussian fits of the histograms give average (μ) and standard deviation (σ) values in each of the three directions. **d-f**. Bar plots of % of fractions that has bone-marker shifts in <0.3 cm, 0.3 - 0.5 cm, 0.5 - 1 cm, 1 - 1.5 cm, and >1.5 cm for AP, SI and RL directions.

When CBCT was registered to the simulation CT using skeletal anatomy, a visible discrepancy in the location of fiducial markers was noted between the two scans (Figure 2a-c). To evaluate whether the extent of the bone-marker differences was related to the respiratory motion range, the standard deviations of the bone-marker differences for each patient were plotted against the fiducial displacements calculated from the simulation BH CTs for AP, SI, and RL directions. No significant (>0.8) correlation was observed. The distribution of the bone-marker registration differences for all patients was subsequently analyzed (Figure 3a-c). Unlike the respiratory motions measured from the BH CTs, on average, the smallest absolute bone-marker difference was observed in the AP direction (2 mm), followed by 3 mm in both RL and SI directions. The differences were symmetric about the skeletal registration, showing no systematic bias or drift in any particular direction. Each histogram was fit to a Gaussian function. As shown in Table 1, if skeletal landmarks alone were used for image guidance, the probabilities of not covering a portion of the tumor during the treatment using different margin strategies were listed. The frequencies of the bone-marker differences in <0.3 cm, 0.3 - 0.5 cm, 0.5 - 1 cm, 1 - 1.5 cm, >1.5 cm range were plotted in Figure 3d-f in three motion axes, with the majority (>90%) of the fractions having the bone-marker difference less than 1 cm.

**Table 1:** Probability of not covering the entire GTV (%)

**Method**	**RL**	**AP**	**SI**	**Total**
Registration using skeletal landmarks alone	69%	4.6%	39%	82%
Strategy I (PTV = ITV + 3 mm)	32%	0.3%	20%	46%
Strategy II (PTV = GTV + 1.5 cm)	0.3%	0%	3.6%	4%

**Figure 4:**
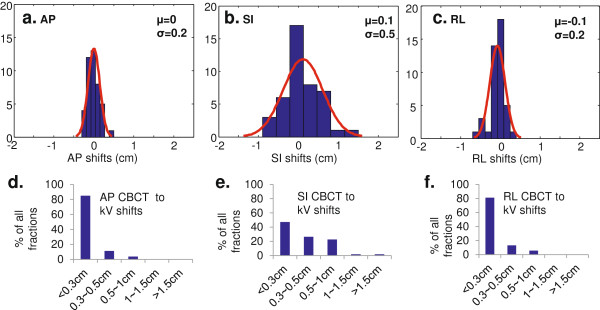
**Statistics for CBCT to kV shifts. a-c**. Histograms of differences between CBCT and kV 2D images for AP, SI, and RL directions, respectively. Gaussian fits of the histograms determine the average (μ) and standard deviation (σ) for each of the three directions. **d-f**. Bar plots of% of fractions that has CBCT to kV shifts in <0.3 cm, 0.3 - 0.5 cm, 0.5 - 1 cm, 1 - 1.5 cm, and >1.5 cm for AP, SI and RL directions.

Each CBCT acquisition takes about 1 minute, during which time multiple respiratory breathing cycles have occurred. The resultant CBCT image thus is representative of the tumor average position. On the other hand, kV 2D images take less than 1 second to acquire and are snapshots which can occur at any phase of the breathing cycle. As all patients were otherwise well immobilized, the differences in fiducial positions between CBCT and kV imaging are most likely attributable to the patients’ breathing. The kV 2D images were obtained after applying the couch shifts determined from the CBCT registration. To understand the intra-fraction motion of the fiducials during daily treatment, the difference in positional location of fiducials between CBCT and kV 2D images obtained prior to each treatment fraction was determined. An example of kV orthogonal images matching to the fiducial contours derived from DRR was shown in Figure 2d-e. The standard deviation of CBCT-kV 2D difference for each patient was plotted against the breathing motion range evaluated from the BH CTs. A weak correlation was noted in the AP and RL directions, but not in the SI direction. The positional differences between fiducials identified on kV 2D images and CBCT images are shown in histograms of the three motion axes (Figure 4a-c). Note that the intrafractional motion was mainly due to the respiratory motion that was included in the ITV margin, which is in addition to the 3 mm ITV to PTV margin. Each distribution is generally Gaussian and the deviations are symmetric about the average marker position in the CBCT. The average standard deviations of kV 2D-CBCT marker distances are 1 mm AP, 1 mm RL and 2 mm SI. Using the reasonable assumption that the CBCT-kV 2D marker distances are a random sampling of the respiratory motion for the patient cohort combined with Gaussian distribution parameters, the influence of intra-treatment motion on the probability of not covering the entire GTV using different margin strategies were listed in Table 2. The frequencies of CBCT to kV shifts in <0.3 cm, 0.3 - 0.5 cm, 0.5 - 1 cm, 1 - 1.5 cm, >1.5 cm range were plotted in Figure 4d-f in three motion axes, with the majority (>98%) of the fractions having the bone-marker difference less than 1 cm

**Table 2:** Influence of Intra-treatment motion on the probability of not covering the entire GTV (%)

**Method**	**RL**	**AP**	**SI**	**Total**
PTV = ITV	32%	0.3%	23%	48%
PTV = ITV + 3 mm	1.2%	0%	7%	8%

**Figure 5:**
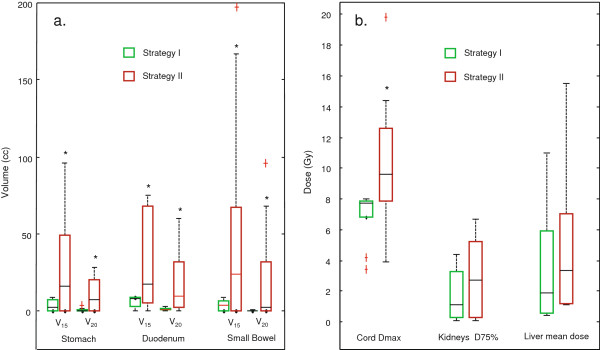
**Box plots of normal tissue doses for two strategies.** All cases satisfied 90 - 95% of PTV receiving the prescription dose. **a**. V_15_ and V_20_ for stomach, duodenum and bowel; **b**. maximum dose for cord, D_75%_ for total kidneys and mean dose for liver. *statistically significant with p < 0.05

All plans evaluated using strategy I met the SBRT planning criteria. All 7 pancreatic and 2 liver patients did not meet one or more of the normal tissue dose constraints using strategy II due to the close proximity of one or more critical organs to the PTV. The liver dose was increased by 36% for the other 2 liver patients that met the SBRT dose constraints with strategy II. Figure 5 shows the dosimetric results for organs at risk, with 90 ~ 100% of PTV receiving the prescription dose. Strategy II significantly (p < 0.05) increased doses for stomach, duodenum, bowel and cord, comparing to strategy I. Strategy II also increased doses to total kidneys and liver, although statistically non-significantly with p value of 0.1 and 0.2, respectively.

## Discussion

4DCT has been widely used for the evaluation of respiratory motion for lung cancer to assess ITV. However, relevance and applicability of 4DCT to abdominal tumors has not been investigated as thoroughly. A recent study from Ge et al. reported that planning 4DCT cannot adequately represent daily intra-fractional motion of abdominal tumors [16]. Study also showed that the maximum intensity projection (MIP) is not useful in determining pancreatic ITV and manual contours are needed [17]. When compared against real-time dynamic MRI images, the deficiency of 4DCT in evaluating motion in abdominal regions is clear [18-20]. For centers without 4DCT, breath-hold CT is one of the limited options left to evaluate tumor motion. It is a valid method to assess abdominal tumor motion [21], and recognized by AAPM task group 76. The use of breath-hold CTs in combination with IV and oral contrast can help clinicians in accurate target definition in both inhale and exhale breath-hold phases. In this study, we have used breath-hold CT to create the ITV, which has the advantages of higher imaging quality and being free from motion artifacts. The accuracy of ITV may be affected by the GTV derived from free-breathing CT which can be motion-blurred, but we expect the effect to be small due to fast 16-row CT acquisition.

Utilization of both CBCT and kV 2D imaging allows pretreatment identification of fiducial markers in both the average and instantaneous positions, respectively. A previous study using kV 2D imaging alone for pretreatment image-guided localization on 9 pancreatic cancer patients reported a motion distribution of 0.2 cm (0.1–0.5 cm), 0.5 cm (0.2–1.5 cm), and 0.4 cm (0.2–0.9 cm) in AP, SI and RL axes, respectively [10]. This distribution is consistent with motion calculated from the fiducial displacement on BH CTs in this study. However, the availability of BH CTs, IGRT with CBCT, and kV 2D imaging lends another dimension to the current analysis. Multiple breathing cycles occur during each CBCT acquisition, so the CBCT images of the markers represent an average position of the markers. The magnitudes of the differences between the bone and marker registrations are a measure of the inter-fractional mobility in the region.

Without using implanted fiducial markers, Shiinoki et al. [22] studied inter- and intra- fractional tumor motion of 15 pancreatic cancer patients using sequential 4DCT during the treatment course. They observed intra- and inter-fractional motion similar to the results obtained here. Mori et al. [23] used high speed 256-row CT to study the intra-fractional pancreatic tumor motion of 6 patients and reported significantly smaller motion in the AP direction than the current work. It is worth noting that these previous studies were unavoidably limited by the intrinsic uncertainties associated with identification and tracking of an organ with low soft tissue CT contrast; the current study provides strong rationale for the use of fiducial markers as a defined surrogate facilitating more accurate tumor and motion assessment.

The significance of this study should be evaluated in the context of SBRT, where the potential for improvements in local control needs to be balanced against possible normal tissue toxicity. Standard chemo-radiotherapy treatment of non-metastatic, unresectable pancreatic adenocarcinoma results in a median survival of 11–12 months, with a high probability of local persistence of disease and poor local control [24]. Whereas use of intensity modulated radiation therapy (IMRT) has reduced normal tissue dose, resulting in improved toxicity rates, it has not allowed clinically meaningful dose escalation using standard fractionation [25]. Local control rates of pancreatic and liver tumors have been dramatically improved following SBRT, although normal tissue toxicities remain high and an impediment to further dose-escalation [26,27]. Concern about the normal tissue toxicity can be partially addressed by improving the geometrical targeting accuracy and confidently reducing treatment margins. In this study, use of skeletal anatomy alignment is shown to result in inaccurate tumor targeting, due to both inter- and intra-fractional tumor motion, and that inaccuracy could not necessarily be accounted for using an ITV. Treatment based on skeletal registration necessitated use of a larger GTV to PTV expansion if the GTV was to be adequately covered. A 1.5 cm isotropic GTV to PTV margin minimally met the coverage requirement, but also resulted in higher normal tissue doses; in most cases, planning constraints were not met and patients would not have qualified for the SBRT. Similarly, a 3 mm ITV to PTV margin, which is typical for SBRT treatment, resulted in grossly inadequate PTV coverage following skeletal registration. Lack of correlation between bone-marker shifts and tumor motion further suggest that the non-isotropic ITV margin expansion may also not be appropriate in this setting, as it does not adequately account for intra- and inter-fraction motion. These results highlight the inadequacy of skeletal registration and further stress the necessity of appropriate soft tissue registration using implanted fiducial markers for SBRT.

The comparison of the pretreatment snapshot 2D kV images to CBCT provided interesting insight into intra-fractional motion. The kV-based shifts weakly correlated with and in some instances also exceeded the magnitude of the ITV margin defined using the BH CTs, suggesting that a portion of the tumor could potentially be outside the ITV for a non-negligible percentage of the time; however, this probability decreased significantly with the addition of a 3 mm PTV margin. One pair of kV images is inadequate to provide a complete motion trajectory. However, we agree that the probability density distribution function of the tumor moving trajectory is Gaussian and kV imaging sampling is random, then the power to determine the mean and deviation of a Gaussian function with 100 samples is approximately 0.85, which is not perfect but useful. Although the significance of this finding is not clear, it implies that intra-fraction motion may not be appropriately accounted for using an ITV calculated from BH CTs. Additionally; our data suggests that without breathing motion management, such as gated or breath-hold treatments, current ITV and PTV margins cannot be further reduced. Certainly, such planning limitations may in turn constrain the ability to dose escalate treatment. To better characterize the pattern of intra-fractional motion, the marker position needs to be continuously monitored through analysis of CBCT projections [28] or by using radiofrequency transponders.

It has been reported that greater tumor respiratory motion amplitudes are correlated to the baseline drift [29] that affects interfractional tumor position reproducibility. The correlation would have considerable implication on the calculation of ITV and PTV margins, e.g. larger ITV margins also warrant larger PTV margins in cases without implanted fiducials. In this study, we investigated the correlation between pretreatment tumor breathing motion amplitude and the magnitude of bone-fiducial registration discrepancy but did not find the correlation.

Whereas the use of implanted fiducial markers and the number of studies investigating their application in the treatment of abdominal tumors is increasing significantly, there are several potential issues related to their use which warrant further study. For example, the distance and spatial relationship between multiple implanted markers and the tumor can potentially change due to treatment and/or disease-related organ swelling or shrinkage. In addition, the distance between the fiducial markers and the tumor may change throughout the breathing cycle due to possible differential motion caused by organ deformation [30]. Finally, although less likely [31], the issue of fiducial migration and/or loss during treatment may occur. Whereas these variables are beyond the scope of this study, they are worth noting when considering an upper-abdominal SBRT program involving EUS-guided placement of fiducial markers.

## Conclusions

Breath hold CT, CBCT and 2D kV images were used to track upper-abdominal tumor motion in 11 patients with intra-tumoral fiducials planned to receive SBRT. Similar to other studies, no morbidity or migration was associated with endoscopic fiducial placement. Tumor motion was greatest in the SI direction. Significant differences in alignment were noted between the skeletal anatomy and the fiducial registration. Interestingly, these differences did not correlate with tumor motion amplitudes as quantified by BH CTs. Following skeletal alignment, a 3 mm PTV margin from the ITV proved insufficient for tumor coverage, while a 1.5 cm margin around the GTV exceeded normal tissue planning constraints. Taken together, these data suggest inadequacy of skeletal alignment and underscore the importance of intra-tumoral fiducials for daily alignment prior to SBRT. Intra-fraction tumor motion, as assessed by snapshot kV 2D imaging, showed only modest correlation with ITV margin calculation, and there was a significant probability that portions of the tumor may not be adequately covered if a 3 mm PTV expansion were not added. These data suggest that further attempts at margin reduction for SBRT treatment of intra-abdominal tumors should incorporate motion adaptive radiotherapy with explicit fiducial tracking or breath-hold techniques.

## Competing interests

The authors declare that they have no competing interests.

## Authors’ contributions

WY, BAF, RR and RT have made substantial contributions to conception, design, data acquisition and manuscript writing; NN, SL, LJ, and KG made substantial contributions to acquisition of data; HS revised the manuscript critically. All authors read and approved the final manuscript.

## Authors’ information

Richard Tuli MD PhD, senior author.
